# Potential of Online Recruitment Among 15-25-Year Olds: Feasibility Randomized Controlled Trial

**DOI:** 10.2196/35874

**Published:** 2022-05-25

**Authors:** Sofie Have Hoffmann, Anna Paldam Folker, Mark Buskbjerg, Marie Paldam Folker, Andrea Huber Jezek, Durita Lyngsø Svarta, Ida Nielsen Sølvhøj, Lau Thygesen

**Affiliations:** 1 National Institute of Public Health University of Southern Denmark Copenhagen Denmark; 2 Centre for Telepsychiatry Mental Health Services in the Region of Southern Denmark Odense Denmark

**Keywords:** recruitment, web based, online, mental health, young people, well-being

## Abstract

**Background:**

Recruiting young people for health and intervention studies by traditional methods has become increasingly challenging. The widespread access to the internet may offer new strategies for online recruitment.

**Objective:**

This study aims to assess the feasibility of online recruitment for a randomized controlled trial evaluating the effectiveness of Mindhelper, an online national youth mental health promotion service. The target group was young Danes aged 15-25 in need of mental health promotion.

**Methods:**

Advertisements for recruitment were set up on Facebook and Instagram. Browser history was collected for a subsample of participants. We compared basic characteristics of participants who completed the baseline survey and those who did not, as well as of participants who completed the follow-up survey and those who were lost to follow-up. The significance of these differences was tested with the Pearson chi-square test.

**Results:**

A total of 560 Danes aged 15-25 were recruited within 1 month (ie, had completed the baseline survey). Among these participants, 356 (63.6%) were at risk of developing depression or stress. The average advertisement price per participant completing the baseline questionnaire was 31 DKK (approximately €4 [US $4.2]). The follow-up survey was sent to 545 participants, of whom 318 (58.3%) completed the survey. No statistically significant differences were observed in baseline characteristics of participants who completed the follow-up and those who were lost to follow-up in terms of gender (*P*=.45), age (*P*=.35), occupation (*P*=.17), cohabitation (*P*=.90), mental well-being (*P*=.26), mental illness (*P*=.44; impact of the illness, *P*=.05), or use of the internet when having a hard time (*P*=.92).

**Conclusions:**

We conclude that it is feasible to recruit young Danes online for a large-scale randomized controlled trial assessing the effectiveness of Mindhelper.

**Trial Registration:**

ClinicalTrials.gov NCT04650906; https://clinicaltrials.gov/ct2/show/NCT04650906

## Introduction

Recruiting participants for intervention studies is increasingly difficult, and participation rates in research projects are generally declining, which may hamper data quality, statistical power, and validity of research findings. Recruitment and retainment of young people with mental health problems, for health and intervention studies, are shown to be especially challenging [1-4].

Most young people are active on social networking sites, being online multiple times a day, which may offer an alternative strategy to recruit young participants. In 2020, 93% of 16- to 24-year olds used the internet on a daily basis in Denmark [5], and 96% of all Danes aged 16-39 were social media users [6], of which Facebook had the largest market share [7].

In recent years, an increasing number of studies have applied online recruitment methods, advertising on, for example, social media platforms, through Google search engine, and by website campaigns. Studies have assessed the feasibility of online recruitment among the general population [8,9], and in specific groups, especially among adolescents, and groups that are considered difficult to recruit by traditional means, for example, men who have sex with men [10-18]. Generally, the results show that the cost per participant recruited online is lower compared with offline recruitment methods [13,18-22], and that it is possible to reach populations who are otherwise challenging to enroll [15,19,23]. Studies find that participants recruited online are younger, more highly educated, have poorer self-rated health, and are more likely to be White and female than representative samples [8,18,20,24-26]. However, a systematic review of studies recruiting for health, medical, or psychosocial research using Facebook showed that the majority (86%) of the studies that examined the representativeness concluded that samples recruited through Facebook had similar representation to those recruited through traditional methods [23]. Further, another systematic review examining studies using Facebook for recruiting participants for health research concluded that recruitment though Facebook was more likely than traditional recruitment methods to result in better representation and improved participant selection among adolescents [18].

The widespread access to and use of the internet further opens opportunities for online interventions. Mental health problems are prevalent among young people both in Denmark and abroad [27,28]. Although young people experience the highest rates of mental health problems of any age group, less than half of young people with mental health problems seek professional help [29-32]. Concerns about stigma and confidentiality, low mental health literacy, and difficulties navigating existing mental health services are among the many barriers to help-seeking among young people [33,34]. Online services may overcome some of the obstacles of help-seeking; however, only few studies have examined the effectiveness of unstructured digital mental health solutions [35-40]. Mindhelper [41] is an online, open-access, self-directed youth mental health promotion service that provides information, self-help tools, and guidance to young people in Denmark. Since January 2019, the service has been freely available and disseminated to young people and youth mental health professionals across the country.

The aim of the study was to assess the feasibility of online recruitment for a randomized controlled trial assessing the effectiveness of a website (Mindhelper) targeting young people aged 15-25 years in need of mental health promotion.

## Methods

### The Intervention: Mindhelper

Mindhelper is a highly scalable, unstructured, multicomponent online mental health promotion service that offers young people information, tools, and support for life problems and mental health difficulties. The site was co-developed (from 2014 to 2017) by the Centre for Telepsychiatry in the Mental Health Services in the Region of Southern Denmark in partnership with young people and 4 Danish municipalities. Mindhelper does not provide psychological or therapeutic treatment. It is designed to provide practical help strategies and tools to support well-being and help-seeking from everyday stressors to more complex mental health issues. The issues range from dealing with family difficulties, depression, and substance use/dependence. Tools and information are derived mainly from the cognitive field, including mindfulness exercises and general strategies to good mental well-being. Mindhelper also offers a supportive outreach service in the form of responding to letters sent by young people. The letters exchanged are published in an anonymized form, so that other young people with similar concerns or worries may benefit from the supportive advice. The website also serves as a national directory to local youth mental health services for further support and help.

The service has been freely available and disseminated across the country since January 2019. In 2020 Mindhelper had more than 1.2 million visits.

### Evaluating the Effectiveness of Mindhelper in a Large-Scale Study

In a large-scale study, we aim to evaluate the effectiveness of Mindhelper, contributing to the much needed evidence base for interventions promoting mental health that target young people [42,43]. This feasibility study was undertaken to investigate whether it is possible to recruit young people with mental health problems via social media platforms, to randomize them to use or not to use Mindhelper, and to keep the intervention and control group separate, although the site is open and freely available online. Further, we aimed to explore whether it is possible to retain the participants in the study over time and to assess the validity of self-reported use of Mindhelper.

### Browser History

To assess whether self-reported questions on online behavior is a valid measure for actual behavior, a subsample of participants, who had given informed consent to access their browser history, was contacted and invited to the National Institute of Public Health (NIPH). Here they met a project employee, who coded the participants’ browser history (in all available browsers) related to the use of Mindhelper for the intervention period from the devices they bought (usually their laptop and mobile phone). All coding was done manually, and thus nothing was downloaded from the participants’ devices. The participants were present during the coding and had full insight into the process.

Because of COVID-19 restrictions, participants were not allowed to enter the NIPH by the end of the recruitment period, and therefore they were guided through the coding process online, meaning that the coding was partly self-reported. Participants who offered access to their browser history were given a gift card for cinema (200 DKK ≈ €27 [US $28.4]) in appreciation for their participation in the study. The financial compensation for participants was given irrespective of their survey responses and browser history. Hence, there was no reason to believe that the gifts impacted the study in any other way than to promote participation in the study.

### The Advertisement Setup

Facebook and Instagram were chosen over other platforms, as the advertisement tools on these platforms enable a more detailed targeting of advertisements than other social media platforms, and because Facebook/Instagram had the lowest cost per eligible contact to participants in a study with a similar aged target group [15]. Advertisements targeted Danish speakers aged 15-25. To allow an easier access to the NIPH, all advertisements further targeted young people living within a 20 km radius from the center of Copenhagen (where the NIPH is located). Age was based on the information listed in the user’s Facebook/Instagram profile, while location was based on the internet protocol address or the address listed on the user’s profile [44].

The advertisements contained a short title (eg, “Help us improve mental well-being among young people”), an image (a photo or drawing of a young male or female looking sad or troublesome), and a main text (eg, “Are you 15-25 years? Help us improve mental well-being among young people. It only requires 2 times 15 minutes of your time”). Examples of the advertisements are displayed in Multimedia Appendix 1. All advertisements were Dynamic Creative Ads, where Facebook’s/Instagram’s algorithms automatically combine title, images, and main text to run based on advertisement performance and the cost per click. In this process advertisements are placed within *advertisement sets*. For this study, 4 sets of advertisements were constructed with a total of 6 advertisements.

Advertisers can choose to be charged per click on the advertisement, or each time the advertisement is displayed a certain number of times [44]. We chose the cost-per-click option, as we were interested in people clicking through to our website at the lowest possible cost. The cost per click depends on the current competition between advertisers within the target group, and thus may fluctuate over the recruitment period.

The first advertisement was released on Facebook and Instagram on November 2, 2020. Participants were led from the advertisements to a webpage for the study, where information about the study and data collection was provided. From this webpage, participants could click their way further to the baseline questionnaire, if they had given informed consent to participate in the study. We used the online survey tool SurveyXact [45], which allowed secure collection and data protection.

### Randomization and Surveys

An automatic randomization was set up allocating everyone opening the baseline questionnaire to either the intervention group or the control group.

Questions on participants’ demographics and use of the internet when they were having a hard time were included. Further, multiple scales were included to assess a broad spectrum of mental health. Well-being was assessed by the Well-Being Index [46,47], psychological distress and daily functioning were assessed by the Short Warwick-Edinburgh Mental Wellbeing Scale (SWEMWBS) [48], while intentions to seek help were assessed by the General Help-Seeking Questionnaire—vignette version (GHSQ-V) [49]. As no Danish version of GHSQ-V exists, the scale was translated by the authors: one in the research group translated the questionnaire from English to Danish, while another translated the Danish version back to English (SHH and DLS). In the few instances where there were inconsistencies between the 2 translations, the best translation was discussed until agreed upon by the researchers.

The survey was pilot tested among 5 young informants within the target group through cognitive interviews, to assess how the posted questions were interpreted and to identify potential challenges in responding to the survey [50]. Special attention was paid to (1) GHSQ-V, as no validated Danish version exists; and (2) new questions formulated by the research group to, for example, measure use of Mindhelper. Based on the informants’ comments, wording was changed to ease the reading, categories corrected to better grasp expected answers, and questions left out due to misinterpretations. However, as no major changes were needed to the questions or structure, it was unnecessary to repeat the pilot testing of the revised questionnaire.

Participants were informed of the follow-up survey, asked to provide their contact details (email or phone number), and informed that they would take part in a lottery of gift cards for the cinema (200 DKK ≈ €27 [US $28.4]) when responding to both surveys. All participants who had completed the baseline survey were invited to answer the follow-up survey 1 week after their completion of the baseline survey. They received the invitation via SMS text message and/or email, depending on the information they had provided in the baseline survey. If they did not respond to the survey within 3 days after the invitation, they received reminders via SMS text message or email. The follow-up survey included questions on the usage of Mindhelper in addition to measures of mental well-being.

Participants were all shown the same questions until the end of the baseline survey. The intervention group was provided with information on Mindhelper and an active link to the website, whereas the control group was informed about the follow-up survey by the end of the baseline survey and received information about Mindhelper only when completing the follow-up survey. On December 2, 2020, the baseline survey was closed; thus, all recruitment was completed within 1 month.

### Statistical Methods

We compared basic characteristics of participants who completed the baseline survey and those who did not and used the Pearson chi-square test to assess if differences were statistically significant. Similarly, we compared characteristics of participants who completed the follow-up survey and those who were lost to follow-up and tested the significance of these differences using the Pearson chi-square test.

### Ethical Considerations

The design of this study was guided by the CONSORT (Consolidated Standards of Reporting Trials) statement for pilot and feasibility trials [51], and conducted in accordance with the Danish Council for Independent Research’s ethical guidelines. All participants received comprehensive information about the purpose of the project and terms of participation and provided informed consent to participate before responding to the baseline questionnaire. The study was registered at ClinicalTrials.gov (NCT04650906), legally approved by the Region of Southern Denmark (journal number: 20/55262), and ethically approved by The Research Ethics Committee of the University of Southern Denmark (case number: 20/68029).

### Consent to Participate

It was clearly stated that participation in the study was voluntary, and participants gave consent that their data could be used for research purposes.

## Results

**Figure 1:**
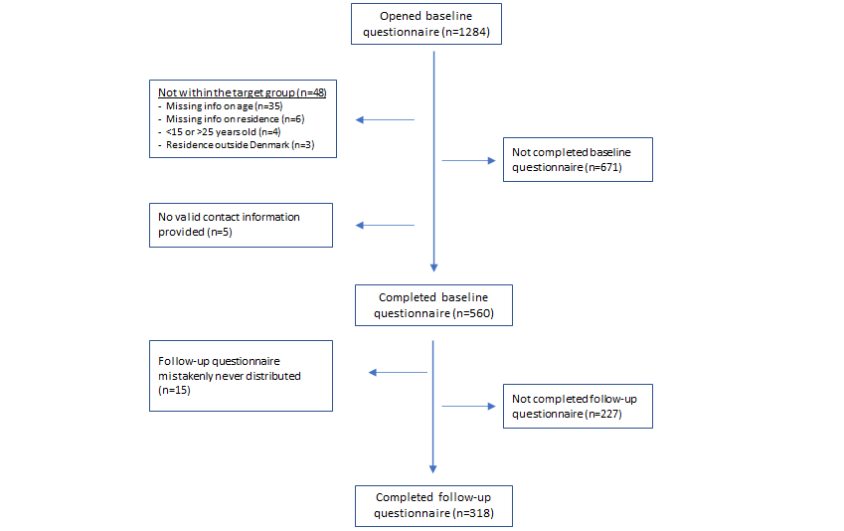
Study flowchart presenting participant recruitment.

The flow of participants in the study population is presented in Figure 1. A total of 1284 participants opened the baseline questionnaire. There were 48 participants who could not be categorized as within the target group, either because they had missing information on age or residency (which were the only mandatory questions within the questionnaire), or because they were younger than 15 or older than 25 years, or not living in Denmark. There were 671 participants who did not complete the questionnaire. Further, 5 participants completed the baseline questionnaire, but did not provide valid contact information, and therefore could not receive the follow-up questionnaire. Thus, a total of 560 participants within the target group completed the baseline questionnaire. All participants completing the baseline questionnaire should receive the follow-up questionnaire; however, 15 participants were mistakenly never invited due to technical errors. A total of 227 participants who received the follow-up questionnaire did not respond, while 318 respondents completed the follow-up questionnaire, and thus a retention probability of 58.3% (318/545) was achieved.

In total, we were able to track 1009 unique clicks to the baseline questionnaire from the landing page. However, the baseline questionnaire had been started 1284 times, thus several clicks to the questionnaire were not tracked. This may occur if participants shared the link to the questionnaire or because of technical issues related to JavaScript. It was predominantly females who interacted with all sets of ads. The average advertisement price per participant completing the baseline questionnaire was 31 DKK (approximately €4 [US $4.2]).

Baseline characteristics of young people who did and did not complete the baseline and follow-up surveys are presented in Table 1. Most of the participants were females, undergoing postsecondary or higher educations, and living together with both parents. More than half of the participants were at risk of developing depression or stress according to the Well-Being Index. Every second participant had at some point been told by a general practitioner or psychologist that they had a mental illness, of whom nearly half (29/52, 56%) were daily affected by the illness during the past 12 months. More than half of the participants used the internet often or sometimes if they were having a hard time.

Participants completing the baseline survey were older, more likely to attend a higher education, and to live apart from their parents than those not completing the baseline survey. However, there was no statistically significant differences between participants completing and not completing the baseline questionnaire regarding gender (*P*=.51), mental well-being (*P*=.39), mental illness (*P*=.17; impact of the illness, *P*=.17), or use of the internet when having a hard time (*P*=.31).

The 560 participants who completed the baseline survey were equally randomized to the control (n=280) and intervention group (n=280), and no baseline differences were observed between the 2 groups (data not shown).

The follow-up survey was sent to 545 young people, and 318 (58.3%) completed the survey. No statistically significant differences were observed in baseline characteristics of participants completing the follow-up and those who were lost to follow-up regarding gender (*P*=.45), age (*P*=.35), occupation (*P*=.17), cohabitation (*P*=.90), mental well-being (*P*=.26), mental illness (*P*=.44; impact of the illness, *P*=.05), or use of the internet when having a hard time (*P*=.92). However, participants who did complete the follow-up survey were slightly older and more likely to be in the risk zone of depression or stress according to the Well-Being Index, compared with those not completing the survey. Furthermore, participants completing the follow-up survey were more likely to have a mental illness and to have been daily impacted by the illness during the past 12 months than those not completing the survey.

During the follow-up period, 21.9% (34/155) of the participants in the intervention group and 3.1% (5/163) of the participants in the control group used Mindhelper (data not shown). The difference in usage between the intervention and control group was statistically significant (*P*<.01).

In total, 49 participants got their browser history coded. However, 3 participants were subsequently excluded as they could not be identified in the survey data, resulting in 46 usable browser histories linked with survey response (Table 2). Overall, when participants in the survey reported that they had not visited Mindhelper, it was consistent with data from the browser history (an agreement between their survey response and their browser history of 94% [31/33]). When participants reported that they had visited Mindhelper, results were less clear (an agreement between their survey response and their browser history of only 64% [7/11]).

**Table 1:** Baseline characteristics of young people who did and did not complete the baseline and follow-up surveys, respectively.

Characteristics		Baseline not completed (n=671)	Baseline completed (n=560)	*P* value	Follow-up not completed (n=227)	Follow-up completed (n=318)	*P* value
**Gender, n (%)**				.51			.45
	Male	87 (13.0)	61 (10.9)		24 (10.6)	37 (11.6)	
	Female	573 (85.5)	488 (87.3)		201 (88.9)	273 (85.8)	
	Other	10 (1.5)	10 (1.8)		1 (0.4)	8 (2.5)	
	Missing^a^	1	1		1	0	
**Age (years), n (%)**				<.01			.35
	15-17	283 (42.2)	135 (24.1)		60 (26.4)	69 (21.7)	
	18-20	139 (20.7)	146 (26.1)		62 (27.4)	79 (24.8)	
	21-23	143 (21.3)	178 (31.8)		63 (27.9)	112 (35.2)	
	24-25	106 (15.8)	101 (18.0)		42 (18.6)	58 (18.2)	
	Mean age (SD)	19.2 (0.13)	20.3 (0.13)		20.1 (0.20)	20.5 (0.17)	
**Occupation, n (%)**				<.01			.17
	Employed	99 (14.9)	85 (15.2)		43 (18.9)	42 (13.2)	
	Unemployed	24 (3.6)	28 (5.0)		11 (4.8)	16 (5.0)	
	Student (primary school)	129 (19.5)	41 (7.3)		18 (7.9)	20 (6.3)	
	Student (postsecondary education)	214 (32.3)	163 (29.1)		71 (31.3)	86 (27.0)	
	Student (higher education)	167 (25.2)	215 (38.4)		74 (32.6)	136 (42.8)	
	Other	30 (4.5)	28 (5.0)		10 (4.4)	18 (5.7)	
	Missing^a^	8	0		0	0	
**Cohabitation, n (%)**				<.01			.90
	Lives with parents (who live together)	228 (35.3)	148 (26.9)		61 (27.6)	84 (26.8)	
	Lives with parents in shifts	52 (8.0)	20 (3.6)		9 (4.1)	9 (2.9)	
	Lives with 1 parent	101 (15.6)	76 (13.8)		32 (14.5)	39 (12.4)	
	Lives with a partner or friend	116 (18.0)	123 (22.4)		47 (21.3)	73 (23.2)	
	Lives in a dormitory	64 (9.9)	71 (12.9)		26 (11.8)	44 (14.0)	
	Lives alone	63 (98)	88 (16.0)		38 (17.2)	49 (15.6)	
	Other	22 (3.4)	24 (4.4)		8 (3.6)	16 (5.1)	
	Missing^a^	25	10		6	4	
**Well-being (WHO^b^ Well-Being Index), n (%)**				.39			.26
	Not in the risk zone (>50 points)	167 (33.2)	198 (35.7)		86 (38.4)	106 (33.7)	
	Risk zone (≤50 points)	336 (66.8)	356 (64.3)		138 (61.6)	209 (66.3)	
	Missing^a^	168	6		3	3	
**Have or have had a mental illness diagnosed by a general practitioner or psychologist, n (%)**				.17			.44
	Yes	52 (7.7)	275 (49.6)		104 (46.4)	166 (52.7)	
	No	48 (7.2)	269 (48.6)		116 (51.8)	143 (45.4)	
	Do not want to answer	5 (0.7)	10 (1.8)		4 (1.8)	6 (1.9)	
	Missing^a^	566	6		3	3	
**Impacted by the mental illness within the past 12 months, among participants with a mental illness, n (%)**				.17			.05
	Yes, daily	29 (55.8)	135 (49.1)		44 (42.3)	87 (52.4)	
	Yes, weekly	16 (30.8)	77 (28.0)		39 (37.5)	38 (22.9)	
	Yes, but less than weekly	7 (13.5)	39 (14.2)		11 (10.6)	28 (16.9)	
	No	0 (0)	24 (8.7)		10 (9.6)	13 (7.8)	
**Use of the internet if having a hard time, n (%)**				.31			.92
	Yes, often	189 (31.2)	148 (26.4)		61 (26.9)	83 (26.1)	
	Yes, sometimes	323 (53.4)	314 (56.1)		125 (55.1)	183 (57.5)	
	No, not at all	80 (13.2)	83 (14.8)		36 (15.9)	44 (13.8)	
	Do not have a hard time	13 (2.1)	15 (2.7)		5 (2.2)	8 (2.5)	
	Missing^a^	66	0		0	0	

^a^Missing entries were not included in the calculations of percentages, and therefore, percentages are not listed for missing.

^b^WHO: World Health Organization.

**Table 2:** Compliance between data from questionnaire and browser history in a subsample of 46 participants.

Comparison between self-reported visits at Mindhelper and visits recorded in browser history		Visited Mindhelper (data from follow-up questionnaire), n (%)		
		Yes	No	Do not know
**Visited Mindhelper (data from browser history), n (%)**				
	Yes	7 (64)	2 (6)	N/A^a^
	No	4 (36)	31 (94)	2 (100)
	Total	11 (100)	33 (100)	2 (100)

^a^N/A: not applicable.

## Discussion

### Principal Findings

In 1 month, 560 participants within the target group and in need of mental health promotion were recruited. Participants completing the baseline survey were generally older than those not completing the survey, and in line with this, a larger proportion attended a higher education and lived apart from their parents. However, no statistically significant differences regarding mental health and well-being were observed between young people with completed and noncompleted baseline surveys.

A retention probability of 58.3% (318/545) was achieved, which is lower than the average probability of 79% observed in a systematic review of engagement of children and young people in digital mental health interventions (ranging from 16% to 100% in the included studies) [52]. However, the average probability covers a wide variety of target groups and interventions, and thus may not be comparable to ours. The study included in the review most like ours targeted Australians aged 16-25 years and assessed the efficacy of an online self-guided app recommendation service aiming to improve well-being [39]. In that study, a retention probability of 50% (4 weeks after baseline) was achieved. Compared with this, a retention probability of 58.3% (318/545) may be acceptable after 1 week. The 318/545 (58.3%) participants who completed the follow-up survey did not differ significantly from those lost to follow-up in demographic characteristics, mental health, and well-being, thus decreasing the risk of selection bias. However, participants who completed the follow-up survey were more likely to be at risk of developing depression or stress, to have a mental illness, and to be daily impacted by the illness than those not completing the survey. This may indicate that we were able to retain participants in need of mental health promotion, and thus to reach the target group of the intervention. The risk of attrition at follow-up will be accounted for in the sample size calculation in the large-scale randomized controlled trial.

We targeted males and females equally; however, we mainly recruited females (488/560, 87.1%). This tendency is in line with other studies recruiting using Facebook, where the majority of the participants recruited have also been females [23]. The advertisement algorithm favors subparts of the target group who have previously interacted with the advertisement, and thus the advertisements were increasingly shown to females during the recruitment process. This may be corrected for in the large-scale effectiveness study of Mindhelper by re-designing the advertisements, oversampling specific groups, or narrowing down the target group for some advertisements (eg, by only including males). The ability to create multiple advertisements targeting different populations, to closely monitor their real-time performance, and to continuously adjust make online advertising a powerful tool for recruiting according to specific demographic requirements. This will help to ensure a broader representation in the study, but will probably also result in a slightly increased price per recruited participant. With an average advertisement price per participant completing the baseline questionnaire of 31 DKK (approximately €4 [US $4.2]), online recruitment proves economically favorable compared with traditional methods. However, one should be aware of the dynamic nature of advertisement algorithms, and the fact that price per click depends on the current competition between advertisers within the target group. Hence, the achieved advertisement price per participant may fluctuate. Further, the advertisement success of future studies may be affected by changing Facebook algorithms and policies.

According to the follow-up survey, 21.9% (34/155) of the participants in the intervention group and 3.1% (5/163) of the participants in the control group had used Mindhelper during the intervention period. Survey-reported online activity was consistent with browser history when participants reported not to have visited Mindhelper, but results were less clear among participants reporting to have visited Mindhelper, due to the relatively few observations and low overall usage of Mindhelper. However, our results indicated that it was possible to ensure that very few participants in the control group used the website, although the website is open and freely available.

A more active and persistent encouragement of participants in the intervention group is needed to increase the usage of Mindhelper. Currently, the most frequent user flow of Mindhelper is people entering the site from diagnostic and research search phrases in search engines (ie, pull marketing). Recruiting through online advertisement flips the user flow (push marketing). Hence, different measures will be applied in the large-scale effectiveness study of Mindhelper to improve the motivation for interaction with the site. Throughout the intervention period, automated series of emails/SMS text messages introducing participants to learn more about the site, and specific series offering advice on improving mental health (eg, evidence-based advice on self-care) will be applied to remind the intervention group of the site’s possibilities.

### Strengths and Limitations

In 1 month, 560 eligible participants within the target group were recruited and thus, we succeeded in recruiting a large study population within a short timeframe. The large study population decreases the impact of nonsystematic errors and improves statistical power. The short recruitment period decreases the risk of other things occurring simultaneously that potentially impact recruited participants and the explored associations.

Some limitations of the study need to be considered. Exposure to the advertisements depended on having a profile on Facebook/Instagram, and the users supplying their correct age in their profile, other users’ interaction with the advertisement (as the advertisement algorithm favors subparts of the target group who have previously interacted with the advertisement), and time spent on Facebook and Instagram. If systematic differences exist between those who were exposed to the advertisements and those who were not, this may give rise to selection bias. Further, there may be considerable differences between those who click on advertisements in Facebook/Instagram and those who do not. Similarly, if young people volunteering to participate in the study vary systematically from those who did not open the survey, self-selection bias may be an issue. As no information was gathered about participants who did not begin the baseline questionnaire, this bias cannot be excluded.

Since recruitment was completed within 1 month, no information was retrieved on longer-term trends in recruitment rates, which may diminish over time. This could be an area for future research.

Young people may access the internet daily from several devices, and they may not have brought all these devices to the NPHI for coding. Additionally, the participants may occasionally browse in incognito mode, and if they did while visiting Mindhelper, this will not show up in the browser history. Therefore, a participant might have actually visited Mindhelper, but it will not show up in his/her coded browser history. This inaccuracy is likely to be highest in the intervention group, as the control group was not provided with information about Mindhelper, and thus the use of Mindhelper may be underestimated in the intervention group.

We were also unable to track all activities from the study website to the questionnaire due to technical issues. In the large-scale effectiveness study of Mindhelper, we will seek to implement the survey directly on the website, which will give us more precise usage data.

Based on the results from this feasibility study, we conclude that it is possible to assess the effectiveness of Mindhelper in a randomized controlled trial and to recruit participants online via social networking sites.

## Appendix

Multimedia Appendix 1 Examples of advertisements.

Multimedia Appendix 2 CONSORT EHEALTH Checklist (V 1.6.1).

## Abbreviations

CONSORT: Consolidated Standards Of Reporting Trials
GHSQ-V: General Help-Seeking Questionnaire—vignette version
NIPH: National Institute of Public Health
SWEMWBS: Short Warwick-Edinburgh Mental Wellbeing Scale
