# Supplementary material for: Potential of Online Recruitment Among 15-25-Year Olds: Feasibility Randomized Controlled Trial
Source: JMIR Form Res. 2022 May 25;6(5):e35874. doi: 10.2196/35874 (PMC9178448; doi:10.2196/35874)
Supplement: Multimedia Appendix 1 [file formative_v6i5e35874_app1.docx]

# Appendix 1: Examples of advertisements.

In this appendix, examples of advertisements are presented, in their original form and language, to give the reader an insight in the design. All ads were Dynamic Creative Ads, where Facebook's/Instagram’s algorithms automatically combine title, images, and main text to run based on advertisement performance and the cost-per-click.
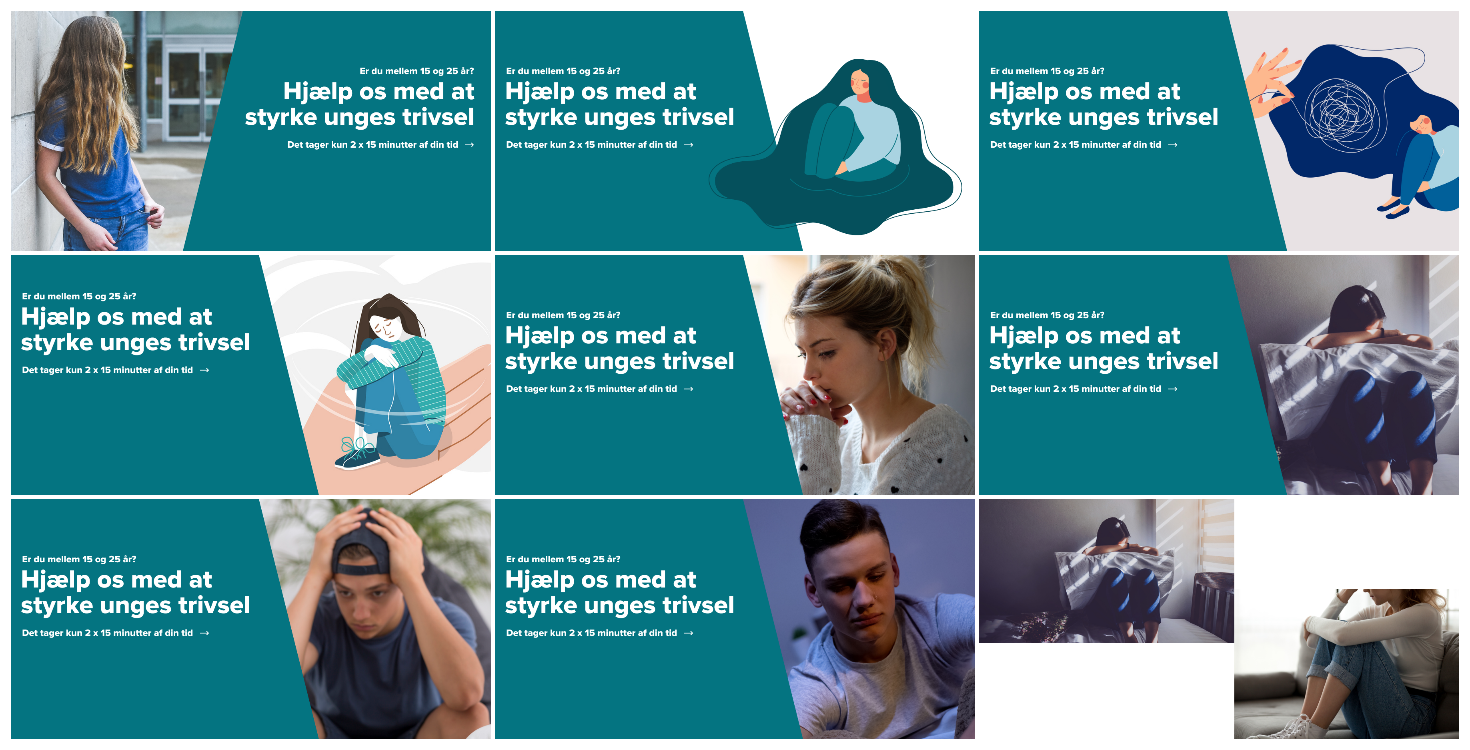
*Examples of adds shown on Facebooks.*


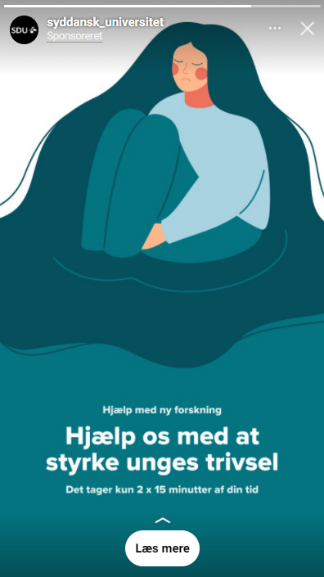

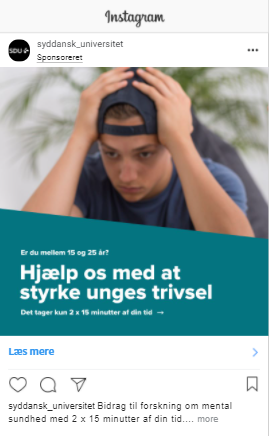


*Examples of ads shown on Instagram. The picture to the left is from the story function on Instagram, while the one to the right is from the explore function.*
